# Uterine rupture in a term pregnancy after a previous uterine artery embolization to manage a large fibroid. A case report

**DOI:** 10.1016/j.crwh.2023.e00551

**Published:** 2023-09-29

**Authors:** Heba Abu Saleem, Nouf Albalwi, Lina Ba'Abbad

**Affiliations:** a Department of Obstetrics and Gynaecology, King Abdullah Bin Abdulaziz University Hospital, Saudi Arabia; b Department of Obstetrics and Gynaecology, Faculty of Medicine, Princess Nourah Bint Abdulrahman University, Saudi Arabia

**Keywords:** Uterine artery embolization, Ruptured uterus, Pregnancy, Laparotomy, Fibroid, Management

## Abstract

Uterine artery embolization (UAE) is an effective minimally invasive alternative to surgery for the treatment of symptomatic uterine fibroids. Uterine rupture is an obstetrical emergency that requires early diagnosis and prompt management to improve perinatal and maternal outcomes.

A 33-year-old woman at 37 weeks of gestation who had had previous two uncomplicated vaginal deliveries at term presented with abdominal pain and rupture of membranes. The patient had undergone UAE for the management of a large anterior wall uterine fibroid three years prior to conception. Vaginal examination revealed cord prolapse and ultrasound showed negative fetal heart. Intrauterine fetal demise with cord prolapse was diagnosed. After admission the patient developed vaginal bleeding and features of hypovolemic shock. Urgent laparotomy revealed a ruptured uterus with a large hemoperitoneum and dead fetus in the abdominal cavity.

Obstetricians should be attentive to the possibility of a spontaneous uterine rupture in pregnant women who have previously undergone UAE for the management of a uterine fibroid.

## Highlights

• Uterine artery embolization (UAE) is an effective minimally invasive alternative to surgery for the treatment of symptomatic uterine fibroids in women who want to preserve their uterus without surgical scar.
• Uterine rupture is a rare and often catastrophic obstetrical emergency that is associated with high maternal and fetal morbidity and mortality.
• Obstetricians should be attentive to the possibility of spontaneous uterine rupture in pregnant women who have previously undergone UAE for the management of a uterine fibroid.
• Contraceptive advice and psychological support are fundamental in the postoperative management of these patients.

## 1 Introduction

Uterine artery embolization (UAE) was first reported in 1995 as an effective alternative to surgery for the treatment of symptomatic uterine fibroids in women who want to preserve their uterus without surgical scars [1]. A Cochrane systematic review compared UAE to surgical approaches (hysterectomy and myomectomy) in the management of uterine fibroids, and concluded that patient satisfaction rates were similar; however, UAE was associated with shorter hospital stay and earlier resumption of daily activities [2]. UAE was associated with a higher incidence of minor short- and long-term complications, hospital readmissions, and surgical intervention rates within five years after the procedure [2]. Furthermore, pregnancy-related complications following UAE including miscarriage, preterm deliveries, higher cesarean section rate, abnormal placentation and postpartum hemorrhage have been reported [3].

Uterine rupture is a rare and often catastrophic obstetrical emergency that is associated with high maternal and fetal morbidity and mortality and is usually related to previous uterine injury from surgery such as myomectomy or cesarean section, uterine instrumentation or trauma [4].

A recent meta-analysis by Akhatova et al. [5] comparing obstetric outcomes after minimally invasive treatments for uterine fibroids showed that, from a total of 250 pregnancies, following UAE no cases of uterine rupture were reported.

Here we report a case of unscarred uterine rupture at term pregnancy in a patient who previously had UAE for the management of a symptomatic uterine fibroid.

## 2 Case Presentation

A33-year-old woman at 37 weeks of gestation in her third pregnancy presented to the labor and delivery department with watery vaginal discharge associated with moderate abdominal pain that had started 5 h earlier. This pregnancy was spontaneous and the patient had had regular antenatal care and a smooth, uneventful course. She had had two previous uncomplicated term vaginal deliveries.

The patient had a history of UAE for a symptomatic intramural uterine fibroid measuring 15 × 9 × 13 cm located in the anterior wall of the uterus three years prior to conception, after which a marked regression in the fibroid size was documented by follow-up scans.

Clinical examination showed normal vital signs and abdominal tenderness. Vaginal examination revealed a 3 cm dilated cervix, rupture of membranes with clear liquor and a cord was felt in the vagina. A bedside ultrasound scan showed absent fetal heart; therefore intrauterine fetal demise with cord prolapse was diagnosed.

One hour after admission, the patient developed tachycardia, hypotension and vaginal bleeding. The clinical suspicion was of uterine rupture. Resuscitation was started immediately and a decision was made for emergency laparotomy.

**Fig. 1:**
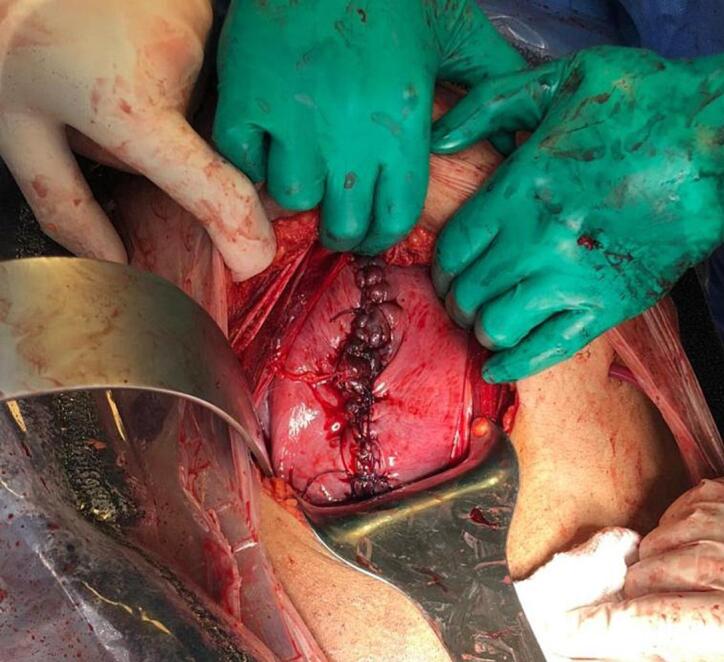
The 12 cm defect in the anterior wall of the uterus repaired using delayed absorbable sutures.

Intraoperative findings showed a dead, normal-looking fetus and placenta in the abdominal cavity, hemoperitoneum of around 1 l of blood and blood clots, and a 12 cm defect in the anterior wall of the uterus extending from the fundus down to the cervix. The defect was repaired using delayed absorbable sutures, and an intraabdominal drain was inserted (Fig. 1).

Intraoperatively, the woman was transfused four units of packed red blood cells and four units of fresh frozen plasma. She was moved to the intensive care unit (ICU), where she stayed for one night. She had uneventful recovery, and was discharged home on the fifth postoperative day.

## 3 Discussion

UAE is a minimally invasive treatment for symptomatic uterine fibroids. The therapeutic effect is thought to arise from post-embolic ischemic changes within the fibroid, resulting in necrosis and volume reduction of the fibroid with subsequent improvement in symptoms such as menorrhagia and pain [6]. The woman in our case had had UAE for the management of a large uterine fibroid, after which she reported a significant improvement in her symptoms and a marked regression in the fibroid size was documented by follow-up scans.

A case series by Carpenter and Walker (2005) evaluating the outcomes of pregnancies after UAE showed 33 (58.9%) of 56 pregnancies had successful outcomes, 17 (30.4%) pregnancies miscarried and 6 (18.2%) were premature. Of the 33 deliveries, 24 (72.7%) were delivered by cesarean section. There were 6 cases of postpartum hemorrhage (18.2%) [3]. In our case the antenatal period was uncomplicated, and the patient had regular antenatal care, with normal investigations and scans results.

The diagnosis of uterine rupture is not often precise. Symptoms and signs may include abdominal pain, vaginal bleeding and features of hypovolemia such as tachycardia and hypotension. However, not all cases of uterine rupture present with these classical features; therefore, it is crucial to keep a high index of suspicion for uterine rupture in patients presenting with some or all of these symptoms and signs regardless of the presence or absence of a prior uterine scar.

Our patient developed vaginal bleeding with hypotension and tachycardia which were suggestive of uterine rupture and ongoing intraabdominal bleeding. A suggested explanation of the intraoperative findings in this case of uterine rupture at the site of the pervious fibroid is the weakening of uterine muscle related to ischemic changes caused by UAE.

Early diagnosis and prompt management are essential to save maternal life. Our management included immediate fluid resuscitation, blood and blood products replacement, aiming for maternal hemodynamic stabilization followed by laparotomy and repair of the uterine defect. Contraceptive advice and psychological support are fundamental in the postoperative management of these patients.

## 4 Conclusion

UAE is an effective treatment for symptomatic uterine fibroids. However, it may be associated with various obstetric complications. Obstetricians should be attentive to the possibility of a spontaneous uterine rupture in pregnant women who have previously undergone UAE for the management of a uterine fibroid.
